# Role of Tumor Microenvironment in Pituitary Neuroendocrine Tumors: New Approaches in Classification, Diagnosis and Therapy

**DOI:** 10.3390/cancers15215301

**Published:** 2023-11-06

**Authors:** Dana Antonia Tapoi, Maria-Linda Popa, Cristiana Tanase, Diana Derewicz, Ancuța-Augustina Gheorghișan-Gălățeanu

**Affiliations:** 1 Department of Pathology, Carol Davila University of Medicine and Pharmacy, 020021 Bucharest, Romania; dana-antonia.tapoi@drd.umfcd.ro; 2 Department of Pathology, University Emergency Hospital, 050098 Bucharest, Romania; 3 Department of Cellular and Molecular Biology and Histology, Carol Davila University of Medicine and Pharmacy, 020021 Bucharest, Romania; ancuta.gheorghisan@umfcd.ro; 4 Victor Babes National Institute of Pathology, 050096 Bucharest, Romania; cristianatp@yahoo.com; 5 Department of Cell Biology and Clinical Biochemistry, Faculty of Medicine, Titu Maiorescu University, 031593 Bucharest, Romania; 6 Department of Pediatrics, Carol Davila University of Medicine and Pharmacy, 020021 Bucharest, Romania; diana.derewicz@umfcd.ro; 7 Department of Pediatric Hematology and Oncology, Marie Sklodowska Curie Clinical Emergency Hospital, 041447 Bucharest, Romania; 8 C.I. Parhon National Institute of Endocrinology, 011863 Bucharest, Romania

**Keywords:** pituitary neuroendocrine tumor, PitNET, tumor microenvironment, cytokines, growth factors, non-tumoral cells, non-coding RNA, immune checkpoints

## Abstract

**Simple Summary:**

Pituitary adenomas are highly prevalent intracranial neoplasms that can be locally invasive in up to half of cases. Consequently, using the term “PitNETs” (pituitary neuroendocrine tumors) instead of pituitary adenoma is recommended to highlight their potential aggressiveness. This review aims to present the latest research on PitNETs based on transcriptomic findings and discuss the microenvironment involved in their development and progression. A better understanding of the role of various molecules and non-tumoral cells in PitNET pathogenesis will result in improved diagnosis, monitoring, and treatment of these entities.

**Abstract:**

Adenohypophysal pituitary tumors account for 10–15% of all intracranial tumors, and 25–55% display signs of invasiveness. Nevertheless, oncology still relies on histopathological examination to establish the diagnosis. Considering that the classification of pituitary tumors has changed significantly in recent years, we discuss the definition of aggressive and invasive tumors and the latest molecular criteria used for classifying these entities. The pituitary tumor microenvironment (TME) is essential for neoplastic development and progression. This review aims to reveal the impact of TME characteristics on stratifying these tumors in view of finding appropriate therapeutic approaches. The role of the pituitary tumor microenvironment and its main components, non-tumoral cells and soluble factors, has been addressed. The variable display of different immune cell types, tumor-associated fibroblasts, and folliculostellate cells is discussed in relation to tumor development and aggressiveness. The molecules secreted by both tumoral and non-tumoral cells, such as VEGF, FGF, EGF, IL6, TNFα, and immune checkpoint molecules, contribute to the crosstalk between the tumor and its microenvironment. They could be considered potential biomarkers for diagnosis and the invasiveness of these tumors, together with emerging non-coding RNA molecules. Therefore, assessing this complex network associated with pituitary neuroendocrine tumors could bring a new era in diagnosing and treating this pathology.

## 1. Introduction

Pituitary tumors originating in the adenohypophysis are frequently diagnosed neoplasms (10–15% of all intracranial neoplasia), and recent studies have proven that up to 40% of them display signs of invasiveness in surrounding structures [1]. Even if their expansion is limited, these tumors can severely impair the noble surrounding nervous tissue; additionally, the development of certain cell populations can disturb the normal activity of the neighboring secretory cells, ultimately resulting in secondary hypopituitarism. In some cases, the hormonal hypersecretion exerted by the tumoral cells, added to the mass effect, can have a significant impact in reducing the quality of life and increasing mortality among these patients [2].

Traditionally, aggressive pituitary adenomas show signs of invasiveness and tend to relapse after treatment, while pituitary carcinomas must be associated with cerebrospinal or systemic metastases [3]. Despite their high prevalence, pituitary tumor definition, classification, and prognosis are still debated [4]. The 2022 WHO Classification of Endocrine and Neuroendocrine Tumors defines these entities as pituitary neuroendocrine tumors (PitNETs), all of which are considered malignant [5]. Additionally, the WHO recommends using the term metastatic PitNET rather than pituitary carcinoma, as there are no well-established morphological criteria for predicting aggressive and metastatic behavior [6,7,8].

The pituitary tumor microenvironment (TME) affects tumor proliferation, angiogenesis, and invasiveness [9]. Despite the accumulated knowledge of pituitary neoplasia pathology and molecular biology, the exact role of the PitNET microenvironment in tumor progression is still not established. 

The tumor microenvironment consists of immune cells (T lymphocytes, NK cells, macrophage/dendritic cells, and mast cells), stromal cells, blood vessels, and various cytokines and growth factors secreted by immune cells or the tumor itself [10,11]. 

In this context, our review aimed to investigate the roles of inflammatory infiltrate, stromal cells, cytokine, and growth factors in the development of pituitary neoplasms and to assess various molecules as noninvasive biomarkers for tumor growth and invasiveness. 

The novel information obtained regarding key players in dictating the behavior of PitNETS is ultimately intended to guide therapeutical approaches, especially for invasive or relapsing tumors that are not responding satisfactorily to conventional treatment. The most remarkable progress regarding new therapy approaches in recent years in various types of cancers is represented by immunotherapy. In this context, the tumor microenvironment (TME), particularly the immune microenvironment, has been recognized as an important contributor to tumor development, progression, and prognosis and is increasingly studied in brain tumors [12]. Less information has been, however, published about TME in PitNETs, and gaining knowledge in this field could play an important role in choosing the most suitable therapeutical strategy. 

Furthermore, growing evidence shows that inflammatory cells are present in the PitNET microenvironment and, together with the other TME components, could influence tumor evolution [13].

## 2. Evolving Classification of Pituitary Tumors

The 2004 WHO classification of pituitary tumors was mainly based on histopathological characteristics and introduced the term “atypical” adenoma [14]. This definition referred to adenomas that displayed invasive growth, had Ki67 > 3%, and were intensively positive for p53 staining. The actual usefulness of this definition was highly debated because this classification did not provide solid criteria for diagnosing invasive growth. According to the 2004 classification, certain adenomas classified as typical can recur or be resistant to therapy, while some atypical adenomas respond better to treatment [3].

The 2017 edition classifies pituitary adenomas according to cell lineage based on the immunohistochemical expression of pituitary transcription factors. Another significant change was eliminating the term “atypical adenoma”, as it proved useless in clinical practice. This classification does not clearly define aggressive pituitary adenomas but recommends assessing the Ki67 index, radiologic invasion, and growth rate to identify “high-risk” adenomas [15].

The attempt to better define pituitary tumors prompted The International Pituitary Pathology Club to propose another classification in 2017, which regards these neoplasms as neuroendocrine tumors. This endeavor stems from the need for a better label for pituitary tumors characterized by a heterogeneous behavior. As shown previously, pituitary tumors are traditionally considered benign and respond well to treatment. Nevertheless, a significant number of pituitary tumors (up to 40%) can be invasive, resistant to medical treatment, and relapse after surgery. Having considered all these factors, The International Pituitary Pathology Club recommends that pituitary adenomas be renamed pituitary neuroendocrine tumors [1]. This proposal is supported by a recent study comparing 125 aggressive adenomas and 40 pituitary carcinomas. The findings demonstrated that the two pathologies shared some common features, such as similar Ki67 and mitotic index values and similar p53 positivity. Both groups’ most frequent tumor type was corticotroph tumors [16].

Because of these recent findings, the latest WHO Classification of Endocrine and Neuroendocrine Tumors, published in 2022, defines tumors of the adenohypophysis hormone-secreting cells as pituitary neuroendocrine tumors and recommends that the term “metastatic PitNET” be used instead of pituitary carcinoma [5]. Additionally, the classification of PitNETs based on transcription factors remains available (Table 1).

We find this recommendation helpful for a better understanding and, hence, a better therapeutic approach to pituitary tumors. They should not be regarded merely as an endocrine disease but as an oncological pathology with endocrine manifestations. As a consequence, future treatment of pituitary tumors should be holistic, involving various medical specialties. 

Furthermore, the 2022 WHO classification of pituitary tumors also mentions the existence of high-risk PitNETs such as sparsely granulated somatotroph tumors, immature PIT1-lineage tumors, acidophil stem cell tumors, Crooke cell tumors, biochemically non-functional “silent” corticotroph tumors, sparsely granulated corticotroph tumors, and null-cell tumors [5,17,18,19,20,21,22,23].

There is an ongoing effort to better classify PitNETs and stratify the patients using multiomics approaches for both tumoral cells and the immune microenvironment in the hope that it will provide improved prediction of tumor behavior and outcomes and suitable immune therapeutical strategies.

Previous studies have shown that during postnatal life, in the anterior pituitary gland and in pituitary tumors, there are populations of progenitor cells that will differentiate into hormone-producing cell types. These cells are believed to be a possible tumor-initiating population originating from an already partially committed cell [24,25,26].

A recent study that performed transcriptomics and methylomics analysis on pituitary tumors established three cellular origins for PitNETs. The transcriptomics analysis of the non-tumor pituitaries revealed that gonadotrope cells showed high transcriptional levels of NR5A1/SF-1, lactotrope, somatotrope, and thyrotrope cells for POU1F1/PIT1 and corticotrope cells for TBX19. Based on the results, the authors identified three distinct molecular groups of PitNETs according to the transcription factor that drives terminal cytodifferentiation. NR5A1 drives the non-functional tumors (gonadotrophinomas, null cell tumors, and STH and PRL-silent tumors), POU1F1 drives GH, PRL, and TSH tumors, and TBX19 drives ACTH tumors. ACTH-silent tumors have been segregated into two transcriptomic groups, one sharing features with non-functional tumors and another with clinically manifest ACTH tumors.

Several upregulated genes encoding proteins with roles as key molecules in many signaling pathways have been successfully identified in the different types of PitNETs. All these neoplasms share some druggable genes (*NRG1*, *KCNA4*, *NCAM1*, *GRIA2*, and *GRM8*). Changes in the expression of genes that encode proteins involved in the WNT signaling pathway, the estrogen signaling pathway, and the calcium signaling path were identified in non-functional tumors. Tumors secreting GH, PRL, and TSH differentially expressed genes that encode proteins involved in fatty acid metabolism, nitrogen metabolism, and the PPAR and HIPPO signaling pathways. ACTH-secreting tumors showed changes in the genes encoding enzymes involved in the metabolism of drugs, xenobiotics, the renin–angiotensin system, etc. Genes involved in cellular senescence, calcium metabolism, fatty acid metabolism, and immune-related events were usually upregulated in all PitNETs. The analysis also included the identification of non-coding RNAs. The expression pattern of these RNA species is also segregated in the three tumor groups. A hypomethylated state was observed in PitNETs compared to the non-tumorous gland. The methylome analysis identified three methylome patterns corresponding to the three groups described in the transcriptomic analysis [13].

In another study that included 200 PitNET patients, Zhang F. et al. performed an integrative genomics, transcriptomics, proteomics, and phosphoproteomics analysis of these tumors. Genomics data indicate that in the pituitary gland, GNAS copy number gain enhances tumor cell proliferation and may serve as a diagnostic marker for hyperproliferation of the PIT1 lineage. Following the proteomic analysis, they proposed a proteomics-based classification of PitNETs. The results showed that tumors overexpressing epithelial-mesenchymal transition (EMT) markers are more invasive. In addition, they identified new potential therapeutic targets, such as CDK6, TWIST1, EGFR, and VEGFR2. Additionally, they identified an association between changes in the JAK1–STAT1–PDL1 axis and immune exhaustion and between the JAK3–STAT6–FOS/JUN axis and immune infiltration, opening a new perspective on the application of immunotherapy for PitNETs [27].

Considering the immunotherapy perspective, a stratification of patients based on the type of immune cells in the TME, their location within the tumor, and their density is also very helpful. Recently, it has been proven that such a classification could predict survival more accurately than the conventional classifications (TNM system), which was revealed for the first time in colorectal cancer. From this immune perspective, the tumors are commonly segregated into hot, altered, and cold tumors. Hot tumors are characterized by high T-cell infiltration and checkpoint activation. They are susceptible to immunotherapy, correlating with a better prognosis. In contrast, an absence of T cells characterizes cold tumors that show limited response to immunotherapy, which are usually associated with poor outcomes [28].

In PitNETs, this immune approach was recently highlighted by Zihao W. et al., who investigated the immune profile of 259 pituitary adenomas using transcriptomic data. This study resulted in a novel classification of these tumors in three clusters based on tumor-infiltrating immune cells (TIICs) and immune checkpoint molecules (ICMs). Cluster 1 exhibited upregulation of CTLA4/CD86 and cluster 2 expressed high levels of PD1/PD-L2, with both clusters revealing a hot immune microenvironment, predicting a high degree of immunotherapy responsiveness. Cluster 3 presented an overall cold immune microenvironment, showing little promise for immunotherapy [2].

## 3. Tumor Microenvironment in PitNETs

### 3.1. Non-Neoplastic Cells in PitNETs

Several cellular types were identified in the tumor microenvironment of PitNETs, including immune cells, tumor-associated fibroblasts, and folliculostellate cells. 

#### 3.1.1. Immune Cell Infiltrate

Tumor-infiltrating lymphocytes have been described in all types of PitNETs, regardless of their size or hormone profile, and linked with disease recurrence [29]. Lu J-Q et al. demonstrated that both CD4+ and CD8+ lymphocytes are present in PitNETs but are significantly more abundant in somatotroph tumors [12]. Some authors reported increased CD8+ lymphocytes in invasive PitNETs [30,31]. Interestingly, CD8+ lymphocytes appear under-expressed in invasive or first-generation somatostatin analog-resistant somatotroph PitNETs [32,33]. In this respect, Marques P. et al. showed that a low CD8:CD4 ratio is associated with increased tumor cell proliferation rather than an absolute decrease in CD8+ cells. Additionally, they showed that macrophages are the most abundant immune cells in PitNETs but failed to correlate these cells with cavernous sinus invasion [34]. On the contrary, Lu J-Q et al. correlated increased tumor-associated macrophages (TAMs) with tumor size and invasiveness [12]. Similar results were also reported by other authors who demonstrated that the number of macrophages is significantly higher in invasive non-functioning PitNETs [31,35] and in invasive gonadotroph Pit-NETs compared to non-invasive ones [36]. CD163+ macrophages are the most important subpopulation in PitNETs, which could be explained by the high concentrations of M2-polarising cytokines found in these tumors [34]. TAMs stimulate invasiveness through M2 polarization via the mTORC2 and ERK signaling pathways [37]. M2 macrophages can also promote neoangiogenesis in pituitary tumors [38].

A 2023 study showed that M2 macrophages are increased and correlated with tumor volume in PIT1 lineage tumors [39]. Additionally, M2 macrophages are associated with invasiveness in various subtypes of PitNETs [30]. Furthermore, TAMs have also been linked to enhancing epithelial-to-mesenchymal transition and thus stimulating the invasiveness in PitNETs [40].

#### 3.1.2. Tumor-Associated Fibroblasts

Fibroblasts represent resting mesenchymal cells that can become activated under certain stimuli to promote physiological processes, such as wound healing, and pathological processes, such as tumor progression. Tumor-associated fibroblasts (TAFs) are aberrantly activated under the effect of growth factors and cytokines produced by neoplastic cells [41]. Consequently, TAFs secrete various molecules involved in extracellular matrix remodeling and tumor growth, invasiveness, metastatic spread, and resistance to therapy [42,43,44,45]. In the context of increasing evidence of the active role of fibroblasts in cancer, there is still little knowledge about TAFs in PitNETs. Similar to other neoplasms, fibroblasts in PitNETs become activated, and their level of activation is associated with tumor behavior, as TAFs from invasive PitNETs display increased expression of α-smooth muscle actin and VEGF compared to TAFs derived from non-invasive PitNETs or to normal fibroblasts [46]. Marques P. et al. showed that TAFs produce numerous cytokines (CCL2, CCL11, VEGF-A, CCL22, IL6, IL8, and FGF-2) in PitNETs, and some of them are associated with cavernous sinus invasion (e.g., IL6), increased vascularization (e.g., CCL2), or higher proliferation rates (e.g., CCL2) [47,48].

Ben-Shlomo A. has also noted that TAFs secrete TGFβ, FGF2, and other cytokines or growth factors involved in drug resistance and increased inflammation in lactotroph and somatotroph tumors [49]. TAFs can also influence the PitNET microenvironment through interactions with other cell types, as TAF-derived FGF2 was correlated with an increased M2:M1 macrophage ratio [48].

One of the proposed mechanisms for TAF-mediated disease progression in PitNETs is through the release of exosomes. In this respect, TAF-derived exosomal circDennd1b has been linked to cell proliferation and invasion in PitNETs by upregulating ONECUT2 expression [50]. This finding is interesting, as ONECUT2 could become a treatment target in aggressive PitNETs. Furthermore, a better understanding of TAFs’ role in pituitary pathology could bring additional therapeutic benefits. For instance, IFN-γ can inhibit TAF remodeling from a myofibroblastic phenotype towards inflammatory/antigen-presenting TAFs [51]. In addition, pasireotide was shown to inhibit TAF activity, decreasing the levels of TAF-derived cytokines [47,48].

#### 3.1.3. Folliculostellate Cells

Folliculostellate (FS) cells are resident non-endocrine cells in the adenohypophysis, attached by desmosomes and gap junctions, forming networks with each other and with endocrine cells [52,53]. These cells form a heterogeneous cellular population (astrocyte-like, dendritic cell-like, and epithelial cell-like) and are identified by their immunoreactivity for the S100 protein [54]. They may co-express GFAP, SOX10, major histocompatibility (MHC) class II surface antigens, and cytokeratins [25,55,56]. FS cells are mainly associated with paracrine control in hormone synthesis and the secretion of endocrine cells from the adenohypophysis. They are also implicated in phagocytic activity and in the secretion of cytokines and growth factors, including interleukin 6 (IL6), follistatin, bFGF, TGF-β, VEGF, LIF, and MIF, etc. [55,57,58,59,60].

Until now, we have gathered insufficient data regarding the population of FS cells in PitNETs. Their role in the initiation, progression, and invasiveness of PitNETs remains unknown.

Farnoud M.R. et al. discovered a transition zone between the tumor and the peritumoral tissue in pituitary tumors with a higher density of FS cells and basement membrane alterations in adjacent peritumoral tissue. These observations suggest the potential involvement of FS cells in remodeling the basement membrane and tumoral expansion [61].

In 198 of the 286 somatotroph tumors investigated, Voit D. et al. identified that FS cells were closely related to the tumor cells. The authors found higher preoperative mean GH levels in patients with tumors containing sparse or scattered FS cells than in patients lacking FS cells. Unexpectedly, preoperative mean GH levels were lower in patients with tumors containing abundant FS cells than those with scattered or without FS cells. In addition, a negative correlation was found between the density of FS cells and the preoperative mean prolactin levels. These results suggest possible changes in hormonal secretion control in PitNETs [62].

Vajtai I. et al. observed an inflammatory reaction in tumoral tissue mediated mainly by CD4+ T lymphocytes in two prolactinomas and one gonadotroph tumor. In the inflammation point were cells that co-expressed S100 protein and HLA-DR. The authors proposed that an appropriate inflammatory tumor microenvironment may lead an FS cell subset to develop a dendritic cell-like phenotype [63].

In a series of 104 non-functional and functional PitNETs, Delfin L. et al. identified CK-positive FC cells in some gonadotroph PitNETs. These cells also expressed transcription factors of gonadotrophs (nuclear SF1 and GATA), data supporting the transformation of neoplastic gonadotroph cells in FC cells. The occurrence of these cells only in gonadotropic tumors is still unclear [64]. 

Wiesnagrotzki N. et al. showed that PitNETs with cellular co-expression of GFAP and cytokeratin were associated with hormone expression and a lower recurrence rate [65]. Ilie M.D. et al. created a cartography of S100B-expressing cells to characterize their interpatient and intratumoral spatial distribution. The study found few S100B positive cells in PitNETs. Additionally, gonadotroph tumors with a higher proliferation rate had few S100B-positive cells. Additionally, the study revealed interpatient and intratumoral heterogeneity in the spatial distribution of S100B-positive cells in gonadotroph tumors [66]. 

FS cells cannot be considered a therapeutic target until we understand their heterogeneity and functions in PitNETs. New therapeutic perspectives would open up if, as in the pancreas, the FS cells in PitNETs could become cancer-associated fibroblasts (CAFs) [67].

**Figure 1:**
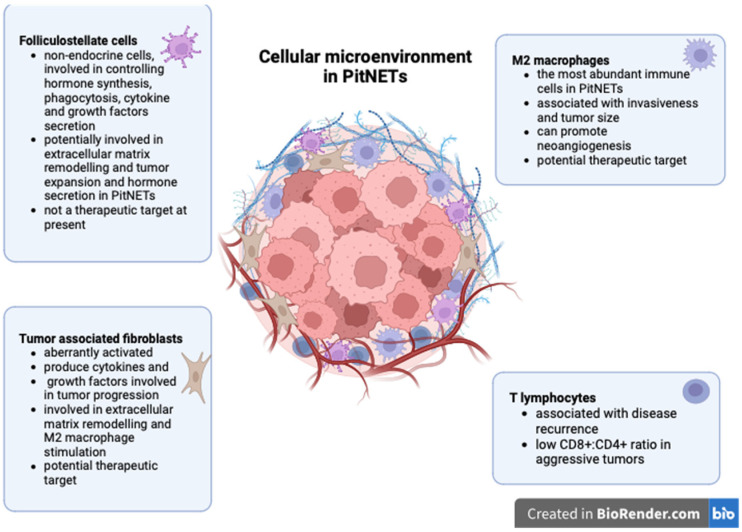
Non-neoplastic cells in PitNETs (created with biorender, Toronto, Ontario, Canada).

The role of non-neoplastic cells in PitNET development is summarized in Figure 1.

### 3.2. Cytokines and Growth Factors Involved in PitNETs

Cytokines are involved in carcinogenesis through multiple and complex interactions with angiogenesis, chronic inflammation, and immune tolerance [9]. 

Due to their pro-proliferative effect and direct correlation to novel vasculature within the tumor, several growth and transforming factors were explored as markers of invasiveness [68,69,70].

#### 3.2.1. Interleukins

Several interleukins (IL1, IL6, IL17) have been studied for their role in pituitary pathology.

Depending on the context, IL6 can be regarded as either a promoter or inhibitor of inflammation and tumorigenesis. IL6 is involved in the pathogenesis of several cancer types and autoimmune diseases and thus has already become a therapeutic target in some of these conditions, such as rheumatoid arthritis [71]. In PitNETs, IL6 may contribute to hormone release, to tumor growth and proliferation, and to the production of angiogenic factors, such as vascular endothelial growth factor-A (VEGF-A) [9]. IL6 is also involved in secretome-induced senescence [72]. Regarding pituitary tumors, IL6 promotes cell growth by paracrine means, whereas by autocrine means, it promotes senescence and prevents malignant transformation [73]. Wang W. et al. demonstrated that IL6 and STAT3 are under-expressed in pituitary tumors [74]. In a 2019 study comprising cell cultures from 24 pituitary neuroendocrine tumors, Marques P. et al. noted that IL6 was highly secreted in 50% of the cases. The concentration of IL6 was significantly higher in nonfunctional tumors than in somatotropinomas. In the same study, 91.7% of the tumors secreted high concentrations of IL8, most of them being nonfunctional tumors. The secretion of both cytokines was increased in tumors with higher macrophage, CD8+ T cell, and neutrophil contents [34]. In addition, they demonstrated that TAFs from pituitary neuroendocrine tumors secrete increased amounts of IL6 and IL8, and the secretion is significantly higher in invasive tumors [47].

Addressing IL6 dysregulation is a well-established therapy in auto-immune disease, but little is known about its benefits for cancer patients. A few trials evaluated the efficacy of IL6 inhibitors in cancer patients, but so far, the results have been unsatisfactory [75,76].

IL17 has been proven to be involved in the development of several cancer types, such as prostate [77], breast [78], and colorectal cancer [79]. On the contrary, IL17 has also been associated with anti-tumoral effects in cancer [80]. Nevertheless, its role in pituitary pathology is scarcely analyzed. Qiu L. et al. first demonstrated that increased serum levels and immunohistochemical positivity are significantly associated with invasive PitNETs [81]. They also argued that IL17 exhibits significantly higher serum levels before surgery, which decrease after surgical tumor removal [82]. A recent study also demonstrated that IL17 serum levels are substantially higher in pituitary tumor patients than in the control group, but they failed to correlate IL17 levels with invasiveness or recurrence [83]. Further research on the role of IL17 in pituitary tumorigenesis may improve treatment options, as target anti-IL17 therapy is gaining considerable interest for various types of cancer [84].

#### 3.2.2. Vascular Endothelial Growth Factor and Other Angiogenic Factors

Vascular endothelial growth factor (VEGF) appears to act as both an inducer and promoter of tumor development by stimulating cell proliferation and migration and increasing vascular permeability.

Different authors demonstrated increased VEGF levels in PitNETs [31,85]. Despite these findings, some pituitary neoplasms can develop without increased angiogenesis and with normal VEGF levels [86,87]. A recent study of 82 pituitary tumors associated VEGF overexpression with GH and PRL-secreting neoplasms. However, the TSH and ACTH secreting tumors exhibited groups of cells positive for VEGF surrounded by VEGF-negative cells. ACTH-secreting PitNETs are often associated with a high proliferation rate that could trigger hypoxia and activate angiogenesis. The same study revealed a positive expression of VEGF in folliculostellate cells, suggesting that this cell type is involved in PitNET genesis [88]. A 2016 study demonstrated that over 70% of the 31 evaluated somatotropinomas expressed VEGF. Nevertheless, its expression was not correlated with tumor size or invasiveness [89]. Other studies demonstrated that both serum levels and immunoexpression of VEGF are elevated in pituitary tumors compared to healthy individuals. Furthermore, VEGF was shown to identify invasive tumors, as it was significantly upregulated in invasive tumors compared to noninvasive ones [90]. He W. et al. showed that by inhibiting VEGF promoters, they obtained a decrease in the invasion rate [85]. A study of 27 PitNETs found a significant association between VEGF-A and VEGFR1 expression and cavernous sinus invasion. These molecules were expressed on endothelial and tumor cells [31].

VEGF was also investigated as a possible predictor for tumor recurrence. VEGF plasma levels were increased in patients before stereotactic radiosurgery and decreased after surgery but remained higher than in control cases [91]. This study offers promising results, proposing the analysis of plasma VEGF as a non-invasive method of monitoring pituitary tumors.

Poveda et al. found increased expression of VEGF-R2 in pituitary tumors but they failed to correlate it with tumor recurrence [92]. Therefore, to some extent, both VEGR-R1 and VEGF-R2 seem to be involved in PitNET pathology.

Xie W. et al. also proved that VEGF mRNA levels are increased in human pituitary tumors. Later, they showed decreased VEGF expression in rat pituitary cells after treatment with cyclin-dependent kinase 5 (CDK5) inhibitors. CDK5 seems to be involved in PitNET development via VEGF stimulation and therefore could be regarded as a target for new drugs against these tumors [93]. Another in vitro study on mouse corticotrope tumor cells demonstrated that matrix metalloproteinase-14 (MMP14) overexpression is correlated with VEGF overexpression and that the inhibition of MMP14 results in a downregulation of VEGF expression. Thus, MMP14 could also serve as a target for future treatment of PitNETs [94].

Additionally, anti-VEGF drugs could be a potential therapeutic option for PitNET patients, particularly in cases of refractory or invasive tumors [95,96]. Di Ieva A. et al. consider VEGF a marker of PitNET aggressiveness and argue that tyrosine kinase inhibitors against the VEGF receptor could be used for treating pituitary tumors [97]. Bevacizumab, a monoclonal antibody against VEGF, has already been used alone or in combination to treat pituitary and carcinomas. Some promising results have been reported, but it is still unclear which regimen is best for pituitary tumors [98,99].

Despite these reports favoring the involvement of VEGF in PitNET development, some studies claim opposing results. For example, Takano S. et al. failed to correlate VEGF mRNA expression with the microvascular density, tumor volume, or tumor histotype [87]. Ilie M.D. et al. claim that VEGF expression is higher in pituitary carcinomas than in pituitary adenomas. Therefore, the involvement of angiogenesis in tumor development is still controversial but appears to play a role in malignant transformation [100]. Yang Q. and Li X. note that VEGF is overexpressed in apoplectic tumors [101], while Gupta P. and Dutta P. state that VEGF is only overexpressed in normal apoplectic pituitary cells, not in apoplectic tumors [102].

Apart from VEGF, several other molecules regulate angiogenesis, such as gremlin, the antagonist of bone morphogenetic protein, which is also involved in cancer angiogenesis. Gremlin was overexpressed in PitNET tissue samples and correlated with CD34-positive vessels [103]. Endothelial cell-specific molecule-1 (endocan) is also considered to play a role in angiogenesis. Thus, a study evaluating 70 PitNETs discovered a correlation between endocan expression and CD34 expression. Endocan expression was also significantly associated with tumor grade, dimensions, and invasiveness [104]. A different study found a significant association between endothelial cell-specific molecule-1 (ESM-1) expression in endothelial tissue and the invasion and size of null cell neoplasms. Positive ESM-1 expression in neoplastic tissue was correlated with tumor size [105].

#### 3.2.3. Fibroblast Growth Factors

Fibroblast growth factors (FGFs) are a family of molecules promoting angiogenesis while stimulating cell proliferation and migration. FGF2 is a member of this family known to stimulate cell differentiation and hormone production in the pituitary gland. Numerous studies have reported that FGF-2 is important in pituitary tumor development. Tanase C. et al. correlated high plasma levels and the immunopositivity of FGF2 with pituitary tumors. Moreover, the highest expression of FGF was recorded in patients with invasive PitNETs. Therefore, serum analysis of FGF may help in investigating aggressive behavior in pituitary tumors [90]. Several studies describe the intricate role of the pituitary tumor transforming gene (PTTG), VEGF, and FGF in PitNET pathogenesis. PTTG stimulates FGF-2, which regulates VEGF expression and promotes angiogenesis [94,101,102]. FGF was also evaluated for recurrence after surgery. Ozkaya H.M. et al. demonstrated that FGF2 is upregulated in patients with pituitary tumors, and the highest levels were found in patients with sphenoid bone invasion. FGF2 levels mostly dropped after surgical tumor excision but remained elevated in recurrent tumors [106].

FGF2 interacts with its transmembrane receptors (FGFRS, of which FGFR-4 is the most prominent one in relation to PitNETs), which are tyrosine kinases responsible for the biological effects of FGF2 [107]. A recent study on 1055 pituitary tumors determined a positive correlation between FGFR4 expression and tumor size. In addition, the highest FGFR4 positivity was found in gonadotrophs, null cells, poorly differentiated Pit-1 lineage tumors, and unusual plurihormonal tumors [108]. Chatzellis E. et al. note that both FGF-2 and FGF receptor 4 are upregulated in PitNETs in general and in invasive ones in particular [3]. Di Ieva also observed that FGF is mainly upregulated in very aggressive tumors, and FGFR4 may be a marker for invasive silent corticotropinomas [97]. Cristina C. et al. associated strong FGF-2 immunopositivity with invasive prolactinomas resistant to dopamine agonists. Furthermore, the expression of the FGF-2 receptor was higher in invasive tumors than in non-invasive ones [109].

Understanding the role of FGF in pituitary tumorigenesis could also improve therapeutic strategies. Anti-FGF therapy is currently being studied for various cancers, but its efficacy has been limited [110].

#### 3.2.4. Epidermal Growth Factor

Epidermal growth factor (EGF) is a mitogenic growth factor involved in cell proliferation and tumorigenesis. It has been shown that EGF is secreted by normal pituitary cells, and it regulates cell growth and prolactin production [107]. It exerts its effects via a tyrosine kinase receptor–EGFR. 

EGFR is usually expressed by pituitary cells, in which it stimulates hormone production. EGFR is also involved in prolactinoma development, promoting tumor growth and prolactin secretion [111]. EGFR is also significantly overexpressed in Crooke cell tumors, a rare and aggressive type [112]. In promoting pituitary tumor proliferation, EGFR was shown to cooperate with ADAM12. Wang J. et al. proved that ADAM12 is associated with cavernous sinus invasion, and silencing ADAM12 inhibits cell proliferation [113]. Later, they demonstrated that ADAM12 promotes cell proliferation and invasion via the EGFR/ERK signaling pathway. Moreover, the authors showed that blockage of EGFR using Gefitinib, a specific inhibitor for EGFR, decreased migration, invasion, and cell proliferation. Therefore, this tyrosine kinase inhibitor, already used in treating various cancer types, should be considered a potential drug for pituitary tumors [114]. 

EGF and its receptor are expressed in somatotropinoma cells, and EGFR is also correlated with PitNET invasiveness [97]. Epidermal growth factor–like domain multiple 7 (EGFL7) is an angiogenic-signaling molecule associated with normal and tumoral angiogenesis. In pituitary neoplasia, EGFL7 expression was considerably elevated in invasive GH-secreting tumors compared to non-invasive ones. In addition, EGFL7 overexpression was correlated with tumor size and recurrence, which means that EGFL7 may be considered a potential marker for assessing tumor behavior and the target of future anti-tumoral treatment [115]. Furthermore, a study on mouse corticotroph tumor cells proved that EGFR inhibitors, already approved for other malignancies, decreased cell proliferation [116].

#### 3.2.5. Tumor Necrosis Factor-α

Tumor necrosis factor-α (TNFα) is a cytokine that has a complex role in inflammation and cancer pathology. In mouse pituitary tumor cells, TNFα upregulates VEGF and MMP-9, thus stimulating hemorrhagic transformation [102]. Similar results have also been noted for human pituitary tumors [101]. However, it is still unclear whether TNFα is a direct mechanism causing apoplexy in pituitary tumors or just a consequence of hemorrhagic transformation and hypoxia.

An immunohistochemical study demonstrated that TNFα was not expressed in normal pituitary tissue. TNFα staining was positive in the neoplastic tissue samples and correlated with invasiveness [117]. A different study found that TNFα is also overexpressed in PitNETS invading the bone [118]. As there are conflicting reports concerning the role of TNFα in pituitary tumorigenesis, this cytokine cannot be considered a potential therapeutic target at present. On the contrary, anti-TNFα treatment for auto-immune conditions may actually increase the risk of cancer, but more studies are needed to confirm this hypothesis [119].

**Figure 2:**
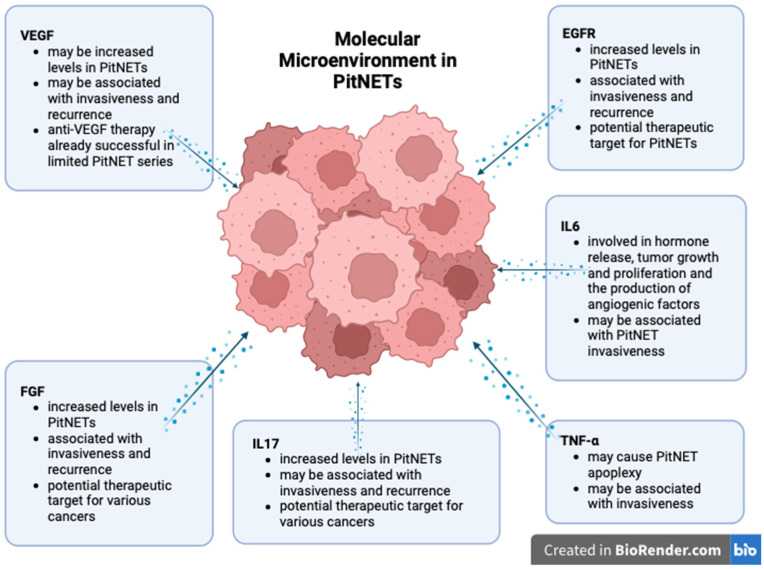
Molecular landscape of PitNETs (created with biorender, Toronto, Ontario, Canada).

The roles of the cytokines and growth factors in PitNET development are summarized in Figure 2.

### 3.3. Immune Checkpoint Molecules

Immune checkpoint molecules (ICMs) regulate immune activity and play an important role in maintaining self-tolerance, controlling the intensity of immune responses. Tumor cells can express immune checkpoint molecules, which suppress the activation of T cells [9]. The most well-known ICMs in cancer are the programmed cell death 1 (PD-1), with its two ligands, PD-L1 and PD-L2, and cytotoxic T lymphocyte-associated protein 4 (CTLA-4), which also has two ligands, CD80 and CD86 [2].

Immune checkpoint molecules play a crucial role in neoplastic progression by aiding tumor cells in escaping the host’s immune system and have been linked to aggressive behavior in PitNETs [120]. In this respect, increased expression of PD-L1 has been found in PitNETs with high proliferation rates [121] or cavernous sinus invasion [31,39]. Despite these promising reports, other authors could not correlate PD-L1 expression with proliferative activity or invasiveness [122]. 

PD-L1 expression may vary depending on the cell lineage of the pituitary tumor. For instance, higher PD-L1 expression has been reported in somatotroph and other PIT-1 tumors in comparison to other subtypes [121,122,123]. 

Additionally, PD-L1 expression may also depend on the age of the patients, as Shi M. et al. reported significantly higher expression in pediatric PitNETs compared to adult cases. Furthermore, PD-L1 was also correlated with tumor recurrence in the pediatric group [124]. 

The general overexpression of PD-L1 in the tumors represents a strategy to protect themselves from the immune response. Therefore, drugs that block the immune checkpoints have been created and have revolutionized the therapeutical field for several types of tumors in recent years [125]. ICM inhibitors, such as anti-PD-L1 monoclonal antibodies, also appear appealing for aggressive PitNETs. 

In addition to M2 macrophage overexpression, PitNETs have been shown to present increased expression of PD-L1, which can be correlated with tumor volume and invasive behavior [39]. Consequently, target therapy anti-M2-cell and immune checkpoint inhibitors could become valuable therapeutic options for patients with aggressive PitNETs [126]. In this respect, recent reports of PitNETs already treated with immune checkpoint inhibitors have shown that this therapy can improve response rates when other treatments have failed [127]. Nevertheless, the efficacy of immunotherapy might not be as high for all types of PitNETs; lactotroph tumors, in particular, may not respond to this treatment [128].

In this context, further studies are required to fully elucidate the relationship between PD-L1 and pituitary neoplasms and establish the benefits of immune checkpoint inhibitor therapy. To date, anti-PD-L1 target therapy has proven successful in murine models with Cushing disease [129] and a limited series of human pituitary carcinomas [128]. More extensive studies should validate these results, and two clinical trials (ClinicalTrials.gov Identifiers: NCT02834013 and NCT04042753) are currently testing immune checkpoint inhibitors in aggressive PiNETs and carcinomas [130].

Therefore, even though the perspective of immunotherapy in PitNETs appears promising, more studies are needed to establish its exact benefits.

## 4. Non-Invasive Biomarkers–Circulating Non-Coding RNAs

Apart from other tumor types, the functional PitNETs benefit from hormone measurements for diagnosis and therapy monitoring. However, the non-functional pituitary tumors, as well as the aggressive ones, require specific biomarkers, as both diagnosis and monitoring currently rely mostly on imagistic techniques. Although there is a constellation of molecules displaying a specific pattern within the tumor or the TME compared to normal pituitary tissue, as discussed above, frequent biopsies of the tumor are not possible, especially considering its challenging location. Therefore, a good biomarker should be easily accessible in a non-invasive manner. Therefore, molecules that show specific behavior in PitNETs and circulating in the blood would be the best candidates, especially since, physiologically, the pituitary is particularly equipped to release molecules into the blood stream, is highly vascularized, and does not present a blood–brain barrier [131]. 

Over the past decade, non-coding RNAs have appeared as popular biomarker candidates because of their stability and tissue specificity. Out of the various types of non-coding RNAs, circulating micro RNAs (miRNAs), long non-coding RNAs (LNCRNAs), and recently also circular RNAs (circRNAs) are being studied as possible biomarkers for pituitary tumors [132]. 

Nemeth K. et al., using a deep next-generation sequencing and qPCR validation approach, reported miR-143-3p downregulation as being directly linked with the surgical removal of FSH/LH-secreting PitNETs, suggesting its role in monitoring the risk of relapse [133]. 

Circulating miRNAs can also serve to discriminate between ACTH-secreting PitNETs and ectopic ACTH-secreting tumors, since the currently used procedure, bilateral inferior petrosal sinus sampling, is invasive and requires trained specialists. Belaya Z. et al. have identified three miRNAs, miR-16-5p, miR-145-5p, and let-7g-5p, that are upregulated in plasma and can pinpoint cases of Cushing syndrome with PitNET origins [134].

Interestingly, all four of these miRNAs found upregulated in plasma could present downregulated levels in pituitary tumor tissue. It can be hypothesized that, in order to permit tumor proliferation, there is a mechanism of excessive dumping of tumor-suppressive miRNAs [135].

Circulating miRNAs were also revealed to indicate invasive PitNETs, independent of the tumor subtype. In a study on mixed PitNET subtypes, the level of miR-200a was increased in invasive pituitary tumors compared with non-invasive ones. Post-surgery, the level of this miRNA decreased in the invasive cases, which did not happen for the non-invasive PitNETs, rendering miR-200a a promising biomarker and a potential therapy target for invasive PitNETs [136].

Compared with miRNAs, there are fewer studies on lncRNAs in PitNETs. Some lncRNAs appear consistently up- or downregulated in tumor samples versus normal pituitary tissue [137]; however, circulating levels have not been sufficiently investigated in PitNETs so far.

Maternally expressed 3 (MEG3) was found to be downregulated in tumoral samples from clinically non-functional PitNETs versus samples from normal pituitaries [138].

H19 is another lncRNA important for the behavior of pituitary tumors that has been especially investigated in lactotroph PitNETs, where it was consistently downregulated. Furthermore, several in vitro and in vivo studies demonstrated that the overexpression of H19 significantly reduced tumor growth [139].

Zhang Y et al., intravenously injected exosomal H19 into a mouse xenograft pituitary tumor model, which reduced the tumor volume and increased its sensitivity to cabergoline, a common therapeutic agent used for the therapy of lactotroph PitNETs [140]. These results emphasize the role of H19 in lactotroph PitNETs, not only as a diagnosis and prognosis biomarker but also as a promising therapeutic agent.

The studies on circRNAs in PitNETs are scarce. There are some promising candidates investigated in nonfunctional pituitary adenoma [141] and in GH secreting PitNETs [142], but their serum fractions have not yet been evaluated [132].

Additionally, there are current strategies to use miRNAs as therapy agents in different tumors, including PitNETs. There are two proposed directions: the restoration of miRNAs that work as tumor suppressors using miRNA mimics and the inhibition of overexpressed “oncomiRs” using various anti-miRNA therapy approaches, aiming to decrease the level of the miRNAs that are overexpressed in the tumor [143,144].

Although it shows very promising results, the implementation of the use of non-coding RNAs as clinical biomarkers is not yet ready to take place, since various challenges still need to be overcome, such as sample collection, storage, and RNA extraction, which still require standardization and optimization. It is especially important that the plasma/serum sample is not contaminated with cells, since the intracellular levels of non-coding RNAs are many times higher than the circulating ones. Nevertheless, the growing interest in the use of non-coding RNAs as non-invasive biomarkers for diagnosis, prognosis, and therapy monitoring is expected to overcome the current challenges. Moreover, the perspective of their use as therapeutic agents should boost these efforts.

## 5. Conclusions

Understanding the molecular mechanisms involved in PitNET development and invasiveness could lead to better management of patients, shedding new light on diagnostic and therapeutic approaches. Nowadays, multi-omic analysis can provide important insight into PitNET behavior, helping in establishing the diagnosis and identifying potentially invasive tumors; it can also be used for the follow-up of the patients in a non-invasive manner. New biomarkers such as non-coding RNA may be integrated into those that are currently available (soluble cytokines and growth factors) based on the patient’s profile. This may promote the use of personalized and target therapy in aggressive and multi-drug resistant PitNETs, such as VEGF and EGF inhibitors and immune therapy with monoclonal antibodies against immune checkpoints. Together with traditional investigations, such as histopathological, radiological, and hormone analysis, the characterization of the PitNET microenvironment represents the beginning of a personalized approach to dealing with these complex and heterogeneous tumors. Furthermore, acknowledging the role of the pituitary tumor microenvironment will lead to improvements in treatment, finally achieving the goal of entirely personalized medical practice.

## Figures and Tables

**Table 1:** 2022 WHO classification of PitNETs.

Tumor Type		Imunophenotype	Transcription Factors and Other Cofactors
Somatrotroph	Densely granulated	GH, α-subunit LMW CK: perinuclear	PIT1
	Sparsely granulated	GH LMW CK: dot-like (fibrous bodies)	PIT1
Mammosomatotroph		GH, PRL, α-subunit LMW CK: perinuclear	PIT1, ERα
Lactotroph	Sparsely granulated	PRL paranuclear LMW CK: weak/negative	PIT1, ERα
	Densely granulated	PRL diffuse cytoplasmatic LMW CK: weak/negative	PIT1, ERα
Thyrotroph		TSHβ, α-subunit LMW CK: weak/negative	PIT1, GATA2/3
Acidophilic stem cell		PRL (predominant), GH (focal and inconstant) LMW CK: fibrous bodies (inconstant)	PIT1, ERα
Mature plurihormonal PIT1-lineage tumor		GH, PRL, α-subunit, TSHβ LMW CK: perinuclear	PIT1, ERα, GATA2/3
Immature PIT1-lineage tumor		GH, PRL, α-subunit, TSHβ LMW CK: focal/variable	PIT1, ERα, GATA2/3
Corticotroph	Densely granulated	ACTH and other POMC derivatives LMW CK: strong	TPIT (TBX19), NeuroD1/β2
	Sparsely granulated	ACTH and other POMC derivatives LMW CK: variable	TPIT (TBX19), NeuroD1/β2
Crooke’s cell		ACTH and other POMC derivatives LMW CK: intense ring-like perinuclear	TPIT (TBX19), NeuroD1/β2
Gonadotroph		FSHβ, LHβ, α-subunit LMW CK: variable	SF-1, GATA2, ERα
Null cell		LMW CK: variable	None
Unclassified plurihormonal tumor		Various combinations	Various combinations
